# Synthetic biology landscape and community in Germany

**DOI:** 10.1016/j.biotno.2021.12.001

**Published:** 2021-12-31

**Authors:** Nicolas Krink, Anne C. Löchner, Hendrik Cooper, Chase L. Beisel, Barbara Di Ventura

**Affiliations:** a Co-founder German Association for Synthetic Biology, Germany; b Previous Steering Committee German Association for Synthetic Biology, Germany; c Steering Committee German Association for Synthetic Biology, Germany; d Advisory Board German Association for Synthetic Biology, Germany; e Max Planck Institute for Terrestrial Microbiology, Germany; f Center for Synthetic Microbiology (SYNMIKRO), Germany; g The Novo Nordisk Foundation Center for Biosustainability, Technical University of Denmark, Denmark; h Helmholtz Institute for RNA-based Infection Research (HIRI), Helmholtz-Centre for Infection Research, Germany; i Medical Faculty, University of Würzburg, Germany; j Signaling Research Centres BIOSS and CIBSS, University of Freiburg, Germany; k Institute of Biology II, University of Freiburg, Germany

**Keywords:** Synthetic biology, Community, Science & politics, Science communication, Research landscape, Germany, Europe, Association

## Abstract

Despite its start in the early 2000s, synthetic biology is still overall perceived as a young discipline. In some countries, such as the US, synthetic biology is academically and industrially established, while in others, including Germany, it is still an upcoming field of research. Issues with funding schemes, commercial translation of technologies, public perception, and regulations need to be addressed to establish synthetic biology as a key discipline of the 21^st^ century. This perspective article reviews the German and European synthetic biology landscape and how the German Association for Synthetic Biology (GASB) is addressing the above-mentioned challenges with its events and community-building activities.

## 1 Introduction

**Fig. 1:**
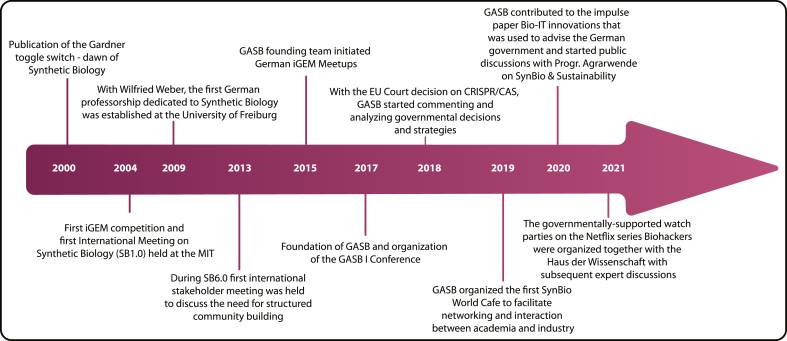
Timeline of synthetic biology in the context of the German Association for Synthetic Biology (GASB).

Since synthetic biology emerged around the year 2000, it has been a growing and prospering discipline that is now transitioning into a mature state^1^^,^^2^ (Fig. 1). The name might be confusing to some, considering that 'synthetic' sounds like the antithesis of 'biological'. Synthetic here does indicate unnatural, something that does not occur in nature; however, interestingly, synthetic biology deals with biological parts. Synthetic is also the adjective of the noun 'synthesis', which indicates the process of creating a whole by combining parts together. As a matter of fact, synthetic biology deals with the creation of novel biological parts and devices that perform a desired new function in a predictable and reproducible manner.

Some of the field's distinguishing features are the variety of approaches being taken and the diversity of scientific disciplines that fall within what we call synthetic biology. The plethora of research conducted under the umbrella of synthetic biology ranges from basic research and proof of concepts to medical, agricultural, and industrial applications fueling the bioeconomy.^3^, ^4^, ^5^, ^6^

Even though synthetic biology might sound to some just a trendy but empty buzzword, the researchers in this field know it holds the potential to solve many of the problems our planet currently faces.^7^, ^8^, ^9^ Beyond skepticism, synthetic biology is met with another, perhaps harder to eradicate, feeling: fear. In many countries, society is ambivalent towards emerging technologies such as synthetic biology because of the potential risks and ethical and societal challenges and impacts they may bring.^10^^,^^11^ Many lay citizens are skeptical towards synthetic biology given its potential usage in warfare, the moral implications of creating or modifying life at will, as well as the potential negative effects of engineered biological devices on health and the environment. Lack of knowledge concerning the basic principles of synthetic biology and its limitations also contributes to the distrust. This results in political and governmental frameworks influencing the advancement of the field.^12^^,^^13^ Thus, to advance synthetic biology, all aspects, questions, and concerns need to be openly discussed and addressed with all stakeholders and interested parties and clear information must be provided in terms understandable by non-scientists.

As a consequence, synthetic biology associations were founded to unite synthetic biologists and form interest groups that facilitate interaction, collaboration, education, technology transfer, or policymaking in many countries and continents. Examples include GASB (German Association for Synthetic Biology, Germany), EBRC (Engineering Biology Research Consortium, US), SynBio Canada, Omic Engine (Greece), AFBS (Association Française de Biologie de Synthèse, France), SINERGY (Singapore Consortium for Synthetic Biology, Singapore), SynBio UK, SynBio Africa, SynBio Asia, EuSynBioS (European Synthetic Biology Society, Europe), and SBA (Synthetic Biology Australasia, Australasia), just to name a few.^14^^,^^15^ Interestingly, the UK sets itself apart from other countries in having established several local synthetic biology associations and communities alongside a national one.^16^

In this communication, we discuss and analyze in the context of GASB the organization and community, the overall landscape, and the status of synthetic biology in Germany. Thereby, we provide an understanding of GASB's history, mission and vision, activities, and efforts towards community building both domestically and internationally.

## 2 Synthetic biology landscape in Germany

In Germany, like in many European countries, synthetic biology research is perceived by the lay public as being performed mostly in the academic environment, because this research is publicly accessible through different channels (lab webpages, funding bodies open repositories, etc.). Companies (if we exclude a few startups) currently rarely mention the term synthetic biology on their official webpages, reinforcing the impression that synthetic biology research takes place predominantly at universities and research institutions. Synthetic biology gained attention in society thanks to the participation of European universities in the worldwide synthetic biology student competition called the international Genetically Engineered Machine (iGEM) competition (see below for more information). European teams regularly perform exceptionally well in this competition, indicating that, despite the US being more advanced in terms of the number of dedicated institutions and funding, scientists in Germany and all over Europe are competitive in terms of ideas and know-how.^17^, ^18^, ^19^, ^20^, ^21^, ^22^, ^23^, ^24^, ^25^, ^26^, ^27^

Since 2009, when the first professorships entirely dedicated to research in synthetic biology were announced (Fig. 1),^28^ many more positions have been established in German universities, and several research clusters, initiatives, and centers were founded. These developments laid the foundation for the advancement of the field in Germany and the establishment of a larger and stronger community. The hubs typically have a dedicated focus, such as synthetic microbiology, signaling, optogenetics, plant synthetic biology, or metabolic engineering, and are a consortium of research groups at local universities and research institutions.^29^, ^30^, ^31^, ^32^ One example is MaxSynBio, a distributed research network by the Max Planck Society and the German Federal Ministry of Education and Research that focuses on understanding the essential mechanisms of cells with the aim to create minimal cells with a bottom-up approach.^33^

Owing in part to the launch of several synthetic biology centers and professorships since 2009, in 2015 10% of all publications involving synthetic biology originated from Germany, a percentage similar to the ones in the UK and China. The US that year made up 50% of publications involving synthetic biology.^34^ This was a five times higher publication output in synthetic biology than that coming from Germany; in general, the US generated four times more scientific and technical publications than Germany, indicating that the US also had relatively higher scientific output in the field of synthetic biology than Germany that year.^35^ According to the research impact in synthetic biology provided by the Australian government, the US has had the highest impact (88744) during the period 2016–2020, followed by China (32320), the UK (23361), and Germany (16847) (the research impact provides an indication of the productivity of a country and was represented by the volume of publications, indicating resources & facilities, and the number of citations indicating the quality).^36^ The number of funding sources as well as the amount of funds allocated to synthetic biology research was reported to be lower in Germany, as in most of Europe in general, than in leading synthetic biology research countries like the US, the UK or China, despite Germany being one of the leading industrial nations.^34^^,^^37^^,^^38^ One factor potentially contributing to the disproportionally lower funding was that the German Research Foundation (DFG), the largest national research funding institution in Europe, did not have synthetic biology, biotechnology, or any other application-oriented life science board or section in their programs.^39^^,^^40^ This, unfortunately, still remains the case.

Synthetic biology should naturally flow into industry, given its high potential for diverse applications. Nonetheless, companies officially working on synthetic biology are underrepresented in many European countries and even more evidently in Germany. Only a few startups exist that use synthetic biology approaches to replace the current chemical production of various compounds with bio-based ones.^41^^,^^42^ The startup ecosystem in Germany, and Europe in general, lack sufficient financing opportunities, business training, and regulatory support, particularly outside of therapeutics. One consequence has been a scarcity of German startups focused on synthetic biology, while in the US and the UK countless synthetic biology startups exist. For example, in 2019, only 1.5% of 6 billion € venture capital in Germany was invested into biotechnology startups; funding for their expansion phase is also lacking, making Germany a difficult place for startups, especially in the life science sector.^37^^,^^43^, ^44^, ^45^, ^46^, ^47^ In the US, on the other hand, between 2016 and 2020, almost 4,000 M $ were invested into synthetic biology, followed by China with ∼200 M $, the UK with 160 M $, France with ∼60 M $, and Switzerland with ∼35 M $, comparing to 10 M $ investment into biotechnology (including synthetic biology) in Germany.^36^^,^^43^ One of the big German industrial players in the life sciences, Bayer, recognized upcoming human challenges and the potential of the so-called "Bio Revolution", including synthetic biology. The company subsequently initiated *Leaps by Bayer* that invested over 1 billion $ since 2015 to tackle these challenges. The program currently supports 23 companies developing health products and 22 dedicated to agriculture. The majority of the supported companies are in the US, a few in Canada, Israel, and Europe, and only one (developing a medical symptoms assessment app) is in Germany.^48^^,^^49^ While a lack of infrastructure for technology transfer and appropriate funding of startups is one aspect justifying the limited number of industrial players in synthetic biology, another factor is the strong focus on basic research rather than applications of the academic research groups in Germany.

Alongside these aspects, synthetic biology still holds a difficult position in Germany. In the initial phase when synthetic biology was emerging, many reports from leading institutions both on a national and European level were drafted evaluating the potentials and risks of this scientific discipline and its effect on research.^50^, ^51^, ^52^, ^53^, ^54^ However, even after two decades of active research in this field, Germany still lacks a synthetic biology roadmap, and the national bioeconomy strategy from 2020 does not mention synthetic biology or related terms at all; in the UK, on the contrary, the first national roadmap was initiated already in 2012.^55^, ^56^, ^57^ As previously mentioned, in general, and especially in Germany, the term synthetic biology is not consistently perceived across society. The term itself is not uniformly defined and often paraphrased, and it is connotated differently through metaphors used in media, making it challenging to transport neutral messages regarding synthetic biology.^58^, ^59^, ^60^ This, in turn, affects the public perception of synthetic biology and, in general, biotechnology and genetic engineering, which then again impacts the national and European regulation of synthetic biology.^61^, ^62^, ^63^, ^64^, ^65^, ^66^

## 3 Community building in synthetic biology

Because most often, researchers transversally enter synthetic biology from many other disciplines (e.g., microbiology, plant sciences, genetics, biophysics, engineering, bioinformatics), they typically remain members of the associations they previously joined.^67^ Many established associations nonetheless formed study groups within their organizational structure to embed and bring together scientists working in synthetic biology. This surely connected various stakeholders of established disciplines to synthetic biology; however, the absence of a domestic or even international cross-disciplinary association did not allow a full exchange between those working in synthetic biology within different disciplines.

Especially the young scientists and students educated in synthetic biology who viewed the field as their primary professional identity were faced with the challenge of not being able to easily connect with other synthetic biologists under one roof. For a long time, the only unifying national (and international) platform for young synthetic biologists was the iGEM competition that provided, besides hands-on experience, a synthetic biology community identity.^68^

During the Synthetic Biology 6.0 (SB6.0) conference in London, a first meeting with stakeholders from different nationalities and disciplines was held to discuss the need for structured community building for the advancement of synthetic biology as a discipline.^68^ Also, during the SB7.0 in Singapore in 2017, German synthetic biologists met to discuss synthetic biology in Germany as well as how to foster interaction of the local SynBio community.^69^ Together with the evolving professional identity originating from iGEM, these exchanges contributed to synthetic biology becoming an independent discipline and to the foundation of synthetic biology associations and organized communities.

### 3.1 iGEM as a community-building event

The iGEM competition is the most renowned worldwide competition in synthetic biology. Students form a team at the beginning of the year, come up with a project idea, which they then realize during the course of the year under the supervision of Ph.D. students, postdocs, and usually at least one PI. Between the end of October and beginning of November, the teams meet during the so-called Giant Jamboree to present their project with a poster and a presentation. Teams are additionally asked to create a website (the iGEM wiki) explaining their project in detail. Participation in the iGEM competition means learning about synthetic biology in a short time, from how to create modular biological parts to discussing ethical issues with the society.^70^

iGEM has a long and highly successful history within Germany. Starting from 2006, iGEM Teams from German universities participated in the iGEM competition. While only the team from Freiburg participated in 2006, 11 teams participated in 2012 and 15 teams in 2017. Because the last iGEM competitions took place during the pandemic, numbers for 2020 and 2021 are not representative.^17^^,^^71^

Undoubtedly, iGEM is globally the priming experience for many future synthetic biologists. One of the core values of iGEM is to establish collaborations to foster exchange, team effort, and community building. To facilitate collaboration-building and to interact with fellow teams in many regions, regional meetups arose, bringing together domestic or continental participating teams before the jamboree in fall every year.^72^ In that spirit, in 2015, the iGEM Marburg team initiated a regional iGEM meetup in Marburg that has become a recurring event (excluding the years of the COVID pandemic) (Fig. 1).^73^, ^74^, ^75^

The success of this meeting platform among iGEMers underlined at the time the lack of a common platform as it provided scientific exchange, discussion, and community building experiences. This eventually resulted in the foundation of GASB that unites both the undergraduate community from iGEM with established researchers and other synthetic biology stakeholders.

## 4 German Association for Synthetic Biology – advancing domestic and international efforts for synthetic biology

### 4.1 Bottom-up community foundation and working framework of GASB

One of the first consortiums dedicated to synthetic biology was SYNBERC (Synthetic Biology Research Center), which was established in the US in 2006 as a multi-university research center funded by the National Science Foundation. It mainly supported established researchers and their teams at various institutions to work on research projects and educate the next generation of synthetic biologists. Additionally, SYNBERC implemented a policy-and-practices group to promote synthetic biology examples to the public.^76^ GASB, which was founded more than ten years after SYNBERC, substantially differentiates itself from the American association.^77^ GASB is a bottom-up, community-driven organization without governmental funding support. It was founded by a group of Ph.D. students and biology students at the Max Planck Institute for Terrestrial Microbiology and the University of Marburg in spring 2017 (Fig. 1). GASB is the common platform for the diverse synthetic biology activities in Germany that serves the community, being a hub for scientific interaction, engagement with public, governmental and industrial stakeholders, educational approaches, overall uniting the community and playing a representative role for the discipline in Germany. Because the research landscape within synthetic biology is very international, the association adopted English as the official language and selected an English name.

GASB engaged with national and international stakeholders from the beginning. For example, GASB is part of the German Life Science Association (VBio) and the European Federation of Biotechnology (EfB). It is also actively engaging with other synthetic biology associations like EUSynBioS, EBRC SynBio Canada, SynBio.Oxford, AFBS, iGEM, and AfteriGEM. Because the community compositions, governmental structures, and obstacles for synthetic biology may differ from country to country, even within the European Union, GASB believes that domestic organizations that internationally cooperate are critical to globally advance and establish synthetic biology as a key discipline for the 21^st^ century.

### 4.2 Agile organizational structure

The organizational structure of GASB is designed to reflect the composition of the synthetic biology community in Germany, which currently comprises mostly university students and early-stage researchers. Community building is a long-term project and takes extensive effort. Thus, it is essential to focus on the next generation of stakeholders in synthetic biology. However, integrating current stakeholders such as leading and established researchers in synthetic biology is also key to the active and diverse community-building efforts of GASB. The association consists of two interacting organizational entities: the steering committee (SC) and the advisory board (AB). The SC is responsible for business operations, including the organization and realization of all events, activities, collaborations, as well as administration and finances. This committee consists of undergraduates and early-stage researchers. As GASB is a registered non-profit association, all SC positions are filled anew each year; they are voluntary and without remuneration. Legally responsible for the association are the head and vice-head of the SC, as well as the financial officer. Beyond these, additional ten members are part of the SC, being responsible for different aspects of the association's work. The AB consists of leading stakeholders in synthetic biology, mainly professors at universities, research centers, and institutions such as the Max Planck or Helmholtz societies. Members are strategically selected to cover the different research areas within synthetic biology. The AB members are appointed by the SC in consultation with the chair of the AB for a period of two years. The AB members closely interact and collaborate with the SC by giving feedback and advice for ongoing projects and activities, are actively involved in the scientific programs of events and in the writing of statements, and contribute their perspective on the field.

GASB is characterized by lean bureaucracy and a lack of entrenched structures. Digital platform solutions facilitate interaction and just-in-time decision-making, allowing GASB to quickly respond to upcoming requests and take action. The AB is requested to promptly provide feedback, allowing moving forward fast—something that additionally characterizes the agile organizational structure of GASB.

The non-profit nature of GASB and the honorary work of all involved contributors allow running cost-effective activities, resulting in financial requirements comparably low to those of other associations. Costs are typically covered by membership fees, donations, and sponsorships dedicated to some of the events. For paid events, attendance fees are proportional to the career stage and different for academia and industry, and they are meant to solely cover costs without generating additional revenue. During the pandemic, all events organized by GASB have been free of charge, as there have been no costs for catering or premises.

The organizational structure of GASB is overall highly inclusive and promotes the engagement of the entire community. Active members are always welcome to support the work of the SC and provide their own ideas, thereby moving the mission and vision of synthetic biology forward.

### 4.3 GASB's community-building efforts through versatile activities and event formats

The synthetic biology community in Germany is diverse in terms of career stage and research focus. However, the values, interests, and needs are common within the community.

**Fig. 2:**
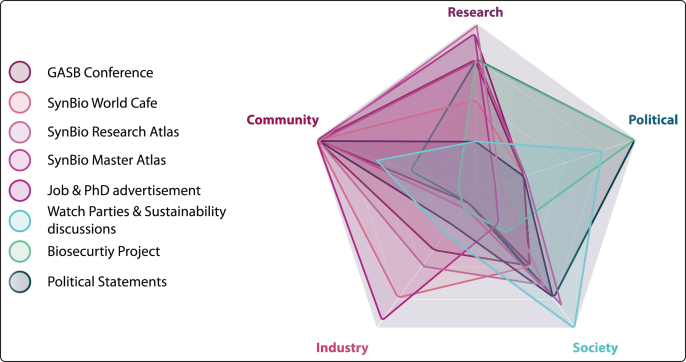
Qualitative representation of GASB’s event formats and activities and their impact on the synthetic biology community, research, politics, society, and industry.

To bring together for the first time all synthetic biologists in Germany and beyond, GASB organized its first scientific conference in the fall of 2017 (Figs. 1 and 2), which has been held annually ever since. Its aim is to foster the interaction and exchange within the community covering the full range of synthetic biology research. The GASB conferences sparked many collaborations among participants and contributed to fostering community spirit. Besides presentations and posters of state-of-the-art research, the conferences feature open discussions in so-called break-out sessions with a wide range of topics such as the current state of synthetic biology in Germany, the perception of synthetic biology by the general public, the European Court decision regarding the usage of CRISPR, visionary talks on the future of synthetic biology, synthetic biology and industrial applications, legal and regulatory requirements for synthetic biology, and synthetic biology in the media.^78^^,^^79^ The conferences are always co-organized at different locations with on-site partners all over Germany.

Talking about the GASB conferences: we wish to share an interesting anecdote because it reflects the state of the synthetic biology community in Germany when GASB was founded. GASB wanted to invite "everyone" working in the field to the first conference (GASB I) and was directly faced with its first challenge. Within the country, the synthetic biology landscape was almost invisible and identifying research groups and stakeholders who publicly identified themselves as synthetic biologists was very challenging.

Therefore, following the first conference, GASB decided to create a synthetic biology research atlas, listing research groups actively working on synthetic biology (Fig. 2). This resource, which is updated every year, turned out to be a real asset for the community: for example, students and early-stage researchers use it to identify labs in which to carry out their research, and journalists find experts for interviews on specific aspects of synthetic biology. A similar atlas is in progress to provide future students in synthetic biology with an overview of study programs offering a specialization in this discipline (Fig. 2).

To foster community building beyond academic research, GASB organizes the SynBio World Cafe, a recurring event focusing on networking, brainstorming, and exchange regarding the future trajectories of synthetic biology between academic, industrial, political, and regulatory stakeholders (Figs. 1 and 2). To facilitate interaction, discussion, and idea exchange in a structured way, the world café format was utilized.^80^ In 2020, GASB joined forces with the High-Tech Forum, a central committee that advises the German Federal Government on implementing its High-Tech Strategy, including biotechnological strategies. Conclusions gathered at this joint World Cafe became part of a recommendation article for the Federal Government (Fig. 1).^81^

### 4.4 GASB's political engagement and outreach

The vision of GASB is to establish synthetic biology as a key discipline of the 21^st^ century and to establish Germany and Europe as a whole as pioneers of the field. GASB also sees its mission in taking a stand for the discipline by engaging with political and governmental stakeholders, critically reviewing developments and raising awareness and interest for the discipline.

Synthetic biology depends, just like other research activities, on public funding, which is in many ways indirectly interconnected with political stakeholders. Especially governmental jurisdictions, treaties, and regulations impact the domestic synthetic biology landscape. Therefore, progressive dialogue with governmental stakeholders is important and has been an integral part of the association's work since its foundation. Starting a dialogue with parliamentarians and parliamentarian groups and reaching out to ministries on federal, national, and European levels was the first cornerstone of the GASB activities. The judgment of the European Court of Justice regarding regulations of genome editing using CRISPR-Cas led to the first official statement of GASB (Figs. 1 and 2), as well as to supporting the open statements to the newly elected European Parliament and Commission of more than 100 European research organizations to enable the potential of gene editing in agriculture and food production.^82^^,^^83^ GASB also commented, critically discussed, and highlighted the interim report of the central commission for biological safety,^11^^,^^84^ the biodefense report by US national academies,^85^^,^^86^ or federal strategy papers such as the national hydrogen and bioeconomy strategies.^55^^,^^56^

Before the German elections, GASB also sent out so-called "Wahlprüfsteine" (Campaigning Questionnaires; Wahlprüfsteine refers to questions that GASB and other interest groups sent to parties participating in federal or state elections to hear the party's opinion and plans regarding specific topics, in this case, synthetic biology), which provided both insights into the party's views regarding synthetic biology as well as its plans for the upcoming term of office.

However, also regulations and policymaking outside of Germany impact the future of the domestic synthetic biology landscape. Therefore, GASB also participated and contributed to events such as the Global Bioeconomy Summit^87^ or the Convention on Biological Diversity.

GASB is, of course, aware of the risks associated with biological engineering and is a strong supporter of safe science and research. Therefore, the association raises awareness among synthetic biologists on topics such as Dual Use Research and counteract potential misuse, resulting from uninformed decisions^88^^,^^89^; members of the SC speak at related conferences, and are for example in the dialogue with military representatives to discuss the risks and potential of dual-use of synthetic biology (Fig. 2).^90^ In addition, GASB is currently developing a program involving universities and other institutions to educate especially early-stage researchers on ethical aspects of synthetic biology.

With its countless applications and thus high potential, synthetic biology has clearly a societal and financial impact. To pave the way for synthetic biology to reach full maturity, associations like GASB need to actively engage in political debates and highlight the impact that governmental decisions can have on the development of a discipline, which is why GASB is moving synthetic biology and the association into the focus of (inter)national policymaking.

### 4.5 GASB in the societal dialogue

Applications of synthetic biology in medicine, the food industry, or agriculture can lead to paradigm shifts that may impact everyday life and, therefore, affect the entire society.^10^ The public perception of biotechnology in general, and especially synthetic biology, is influenced by media and pop culture on topics like engineering life or shaping the perception of science and the field, and not always in a rational and realistic way.^62^^,^^63^ Therefore, interaction with the general public is essential to ensure open discussions at eye-level about the possibilities and impact of synthetic biology on everyday life applications. In order to foster interaction with the general public, GASB is taking several approaches. Since the beginning, GASB has supported iGEM teams in their proactive outreach in the context of Human Practices.^91^

During the pandemic, GASB developed two digital concepts for the general public. On the one hand, GASB partnered with Öko-Progressives Netzwerk (previous: Progressive Agrarwende) to regularly organize online discussions on topics at the interface between sustainability and synthetic biology (Fig. 2).^92^^,^^93^

On the other hand, with the airing of the Netflix series "Biohackers" in 2020, GASB initiated "Watch parties" to jointly watch the series and afterward discuss the scientific accuracy of the story.^94^ For the second season in 2021, GASB partnered with Haus der Wissenschaft to organize watch parties with subsequent expert rounds to discuss science and science communication and the influence of pop culture on the public perception of synthetic biology (Figs. 1 and 2).^95^

GASB is also working towards improving synthetic biology education by targeting educators as multipliers in general public education.^87^

Overall, GASB's collaborative activities are versatile, addressing various aspects of its mission and vision. GASB tries to follow a holistic approach addressing all stakeholders. Eventually, GASB is convinced that synthetic biology can truly advance only through the engagement of the community with the general public, other organizations, the startup community, the established industrial sector, and political stakeholders.

## 5 Conclusion: synthetic biology vision and recommendations

With all its activities, GASB, together with the synthetic biology community, is working towards the establishment of this discipline as a leading player for solving the global challenges of the 21^st^ century. The continuous efforts of the association aim to obtain a national road map for synthetic biology and foster the interaction between synthetic biologists on a global scale. To promote a future sustainable bioeconomy that includes synthetic biology concepts, we recommend the foundation of domestic synthetic biology organizations. These associations should unite on an international level to combine their efforts while locally uniting, shaping, and advancing the discipline, strengthening communities, and addressing domestic needs. Such an international overarching independent organization should provide the frame for communication at eye level and decide on and work on uniting topics. Domestic efforts are required to shape public perception, national regulations, and community building. On the other hand, an internationally united voice of synthetic biologists will gain bigger audiences, impacting the global status and awareness of the discipline. Not only in Germany but also in Europe and globally, synthetic biology still faces problems with public perception, funding, regulation, and commercial translation, that require united efforts to be successfully addressed.

Associations like GASB will make a difference with their broad spectra of activities. By strengthening and uniting the community, political and societal engagement in the name of synthetic biology, these associations will contribute to establishing synthetic biology as a core discipline and thus contribute to solving challenges of the 21st century.

## Author contributions

All authors discussed the scope of the manuscript. NK and AL wrote the manuscript, with input from HC, CB and BDV. HC made the figures.

## Declaration of competing interest

All authors declare that they have no conflicting interests.
